# Hydrodynamics of cruise swimming and turning maneuvers in *Euchaeta antarctica*

**DOI:** 10.1038/s41598-024-76439-1

**Published:** 2024-11-15

**Authors:** Mohammad Mohaghar, Donald R. Webster

**Affiliations:** School of Civil and Environmental Engineering, Georgia Institute of Technology, Atlanta, GA 30332-0355 USA

**Keywords:** *Euchaeta antarctica*, Copepod hydrodynamics, Tomographic particle image velocimetry, Marine biology, Biomechanics, Fluid dynamics

## Abstract

The hydrodynamic disturbance generated by the adult copepod *Euchaeta antarctica* during cruise swimming is quantified. Kinematic results are compared to previous results reported for different *Euchaeta* species. The results reveal a linear relationship between cruise speed and prosome length across *Euchaeta* species, indicating a size-proportional trend that is indicative of a complicated interaction of species size and environmental factors such as fluid temperature and viscosity. The detailed fluid flow measurements using the tomographic Particle Image Velocimetry (tomo-PIV) technique provide insight into copepod cruise propulsion during turning events in comparison to straight motions. During straight swimming, *E. antarctica* demonstrates streamlined flow patterns and reduced vorticity in the near-body fluid shear layer, which is beneficial for sustained motion and energy conservation. In contrast, turning maneuvers are characterized by maximum flow velocities reaching 1.5 times greater values than during straight cruising with increased flow field complexity and enhanced vorticity. The viscous dissipation rate generated in the flow disturbance is also greater during turning events, with the total dissipation rate reaching $\bf3.5-3.8\times 10^{-8}$ W compared to $\bf2.6-2.8\times 10^{-8}$ W during straight cruising. The flow disturbance also generates a hydrodynamic cue that prey may sense in order to avoid the predator *E. antarctica*. For the adult *E. antarctica*, the hydrodynamic cue extends to a volume that is 11–13 times larger than the copepod exoskeleton volume during the straight swimming motion and 22–25 times larger during the turn events.

## Introduction

*Euchaeta antarctica* is a calanoid copepod that plays a key role in the ecology of the Southern Ocean, where its collective biomass is dominant among copepod species^1,2^. This species acts as a voracious carnivorous predator on other copepod species and serves as food for higher planktivores, therefore serving a critical role in the food web of the Southern Ocean^3^. Given the ecological importance, there is significant motivation to improve our understanding of this species, including the biomechanics of its propulsion and hydrodynamic sensory interactions.

Fluid motion in the volume surrounding copepods is key to gaining insight into their biomechanics. Copepods propel themselves through the water via two distinct behavior modes^4–6^. The first mode is a relatively slow swimming behavior that is often described as “cruising”^7^. This mode encompasses a range of propulsion velocities, including a nearly stationary (hovering-like) motion in which copepod appendage stroking induces a laminar feeding current to facilitate suspension feeding, which is effective for a herbivorous dietary habit^8^. Increasing the propulsion speed through the water is accomplished by high-frequency stroking of the feeding appendages and the four or five-leg appendage pairs located along the metasome^9^. The sequential stroking of the appendages located on the ventral side of the copepod generates propulsive thrust as well as a propulsion jet in the copepod’s wake. At moderate speeds, this swimming mode is generally related to predatory feeding and a carnivorous dietary habit, as observed in *E. antarctica*^3^. Variations in this mode may include cruise-and-sink and hop-and-sink behaviors in which copepods temporally alternate between active upward motion and passive downward sinking^4^. The second propulsion behavior mode consists of a high-speed “escape” or “jump”^7^. Typically, the escape mode is evoked due to an external stimulus, such as the presence of a predator, and is directed away from the stimulus^10^ or toward prey^11,12^. This fast mode always involves the rapid stroke of the antennules to induce a quick acceleration, which leads to body and wake vortices^13,14^. For each swimming mode, the interaction of the appendages with the surrounding fluid leads to a unique flow pattern^15–17^.

Quantifying the fluid motion around copepods is also important for sensory ecology considerations^18,19^. Detection of prey and avoidance of predators for many copepods is primarily done via hydrodynamic sensing of fluid disturbances. For instance, hydrodynamic sensing leads to a size selectivity feeding behavior in the adult female stage of *E. antarctica*^3^. There is evidence that spatial gradients of fluid velocity, specifically the fluid strain rate, signal copepods to evoke an escape response to evade predators^20,21^. Alternatively, for a predator perceiving prey, Kiørboe & Visser^20^ argue that the fluid velocity magnitude provides the relevant signal. In either scenario, a pathway to understanding the sensory ability and response is to quantify the fluid flow with high spatial and temporal resolution in order to resolve the rapidly evolving fluid velocity and spatial gradients of fluid velocity surrounding the copepod. It is presumed that copepods have an advantage of being cryptic to prey to facilitate an effective attack, as well as an advantage of being cryptic to predators to avoid being detected.

With an appreciation for the need to understand and quantify the fluid motion induced by copepods, the genus *Euchaeta* presents a fascinating case study. The genus *Euchaeta* ranges throughout the Earth’s oceans. For instance, *Euchaeta rimana* inhabits relatively warm tropical waters^22^. This species is anatomically similar to other species, such as *Euchaeta norvegica*^23^ and *E. antarctica*^1^, that inhabit cooler waters at higher latitudes. While anatomically similar, there are significant differences among these species that are important considerations for the fluid dynamics of propulsion and hydrodynamic sensing. *Euchaeta rimana* is a tropical species with a prosome length of roughly 2.5 mm found in $23 \,^{\circ }$C waters^22^; *Euchaeta elongata* is a temperate species with a prosome length of roughly 4 mm found at $8 \, ^{\circ }$C^24,25^; *E. norvegica* is a high latitude species with a prosome length of roughly 6 mm found at $6 \, ^{\circ }$C^23^; and *E. antarctica* is a polar species with a prosome length of roughly 9 mm found at $0 \, ^{\circ }$C^3^. Hence, quantitative information about the induced hydrodynamic disturbance provides insights into the effects of body size and fluid viscosity (which decreases with temperature and increases with latitude) on propulsion and sensory capabilities.

Previous studies have quantified the fluid motion surrounding *E. rimana* and *E. elongata*^26^ and the CV stage of *E. antarctica*^27^. These studies were limited to a single plane that bisected the specimen using the Particle Image Velocimetry (PIV) measurement technique. Despite being limited to a bisecting plane, the flow measurements provide insight into the cruise and escape swimming modes. For the cruise mode, the quantitative information provided includes the fluid velocity disturbance, strain rate generated in the surrounding fluid flow, and the total viscous dissipation rate. The results reveal a complex interplay between body size, fluid viscosity, and spatial extent of the hydrodynamic signal to prey for *E. rimana* and *E. elongata*. The insights gained are limited by the relatively narrow range of congener sizes studied, as well as the limitations of the planar measurements around a three-dimensional organism.

The objectives of this study are to quantify the hydrodynamic disturbance surrounding a freely moving adult *E. antarctica* during cruise swimming mode consisting of forward motion at moderate speed. The tomographic PIV measurement approach quantifies the full three-dimensional flow field in order to provide unprecedented details about the fluid motion surrounding an adult *E. antarctica*. The results provide insight into the propulsion ability of *E. antarctica* through analysis of the generated flow. Of particular interest are the characteristics of the flow disturbance during a turning event. The measurements also facilitate a comparison of the flow disturbance, vorticity, and strain rate field compared to the smaller *Euchaeta* congeners. Such a comparison is particularly of interest since Svetlichny et al.^7^ note that the scaling of swimming speed and power for propulsion (as a function of the prosome length) deviates for *Euchaeta* species compared to other copepods, perhaps due to the effects of varying fluid temperature and viscosity.

## Materials and methods

*Euchaeta antarctica* individuals were collected from Palmer Deep ($64^{\circ }57^\prime$S, $64^{\circ }24^\prime$W) in the Southern Ocean on-board RV Laurence M. Gould. After collection, animals were held in large buckets of seawater with a salinity of 34.6 parts per thousand (ppt), stored at $0^\circ$C, and transported to the cold room in Palmer Station (Anvers Island, Antarctica; $64^{\circ }46^\prime$S, $64^{\circ }03^\prime$W). Measurements were performed within the two weeks since capture, although the copepods can live for over 3 months in the laboratory. Seawater was obtained from the coastal ocean waters near Palmer Station, filtered, and placed in a glass test tank (10$\times$10$\times$12 cm, W$\times$D$\times$H). The test tank was filled to a height of 10 cm. Within this controlled environment maintained at $0^\circ$C, *E. antarctica* specimens were permitted to swim freely, ensuring natural behavior and accurate representation of their swimming dynamics.

**Figure 1:**
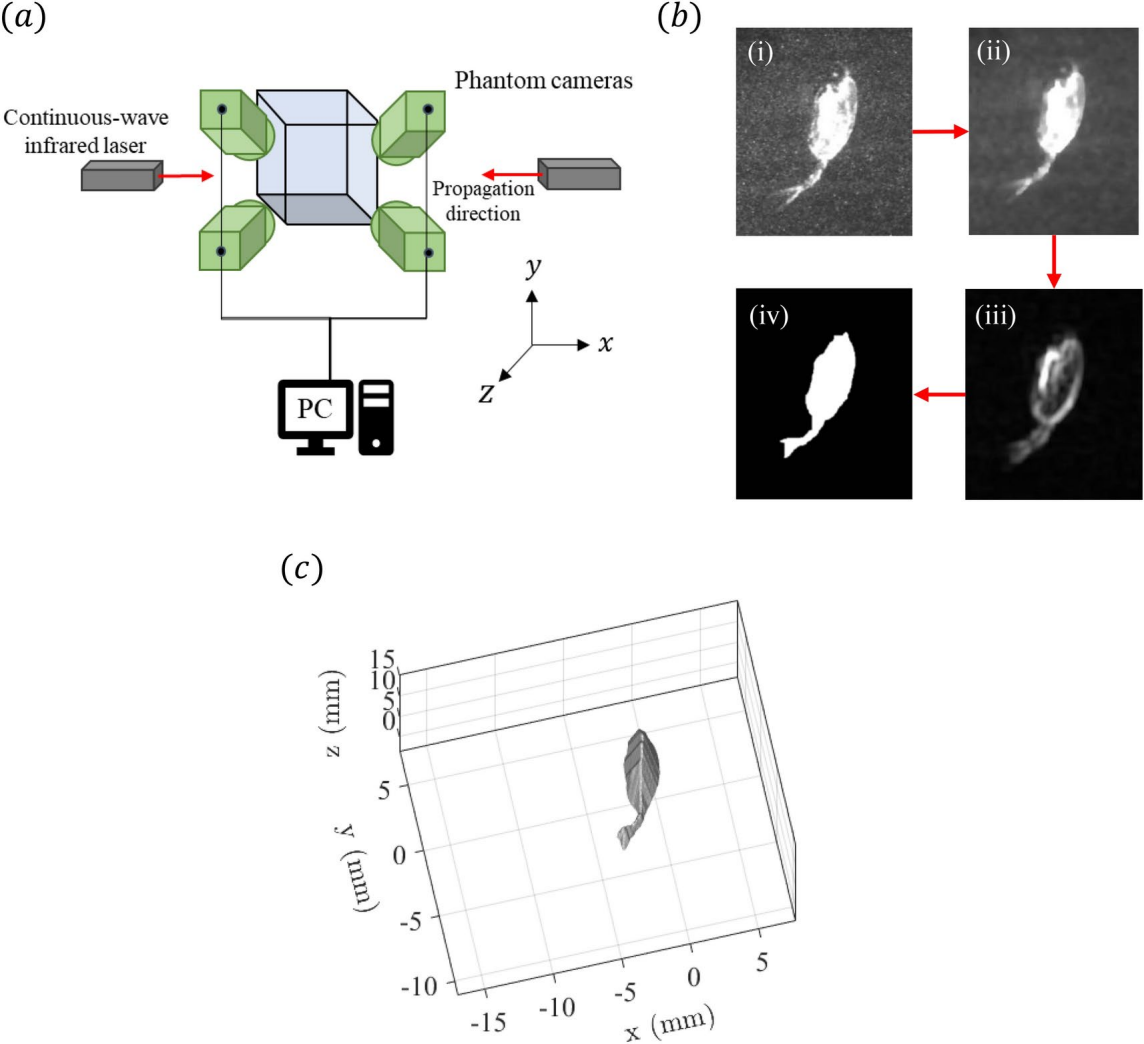
(**a**) Schematic of the tomographic Particle Image Velocimetry (tomo-PIV) system to acquire flow field data surrounding *E. antarctica*. (**b**) Image processing sequence to obtain the silhouette of the copepod required for constructing the visual hull: (i) Original image, (ii) image after applying a median filter to neighboring 5$\times$5 pixels, (iii) image after applying standard deviation filter to neighboring 5$\times$5 pixels, and (iv) image after applying morphological dilation, filling to neighboring pixels, and morphological erosion to create a silhouette. (**c**) An example visual hull representation of the *E. antarctica* specimen (generated with MATLAB).

High-speed tomographic Particle Image Velocimetry (tomo-PIV) was used to measure the volumetric velocity field^14,28^. For illumination, two 7 W continuous-wave infrared lasers (CrystaLaser, Inc.) operating at a wavelength of 808 nm were utilized, which is important to avoid copepod photo-response that happens for illumination in the optical wavelengths^27^. The lasers are strategically placed on either side of the tank to prevent “shadowing” by the *E. antarctica* specimen (shown in Fig. 1a). The lasers created overlapping illumination volumes, thereby ensuring comprehensive coverage surrounding the specimen. The measurement region, illuminated with the lasers, had a length (*x*-axis), height (*y*-axis) and thickness (*z*-axis) of 32, 19 and 13 mm, respectively. Four high-speed cameras (Vision Research Inc. Phantom v210; 1280$\times$800 pixels) were mounted on three-axis geared heads (Manfrotto 400) and synchronized to record at 200 fps. The cameras were angled at approximately $30^{\circ }$ to the *z*-axis and aimed at the measurement volume (Fig. 1a). Each camera was fitted with a Scheimpflug mount to correct for off-axis optical distortion and a 105 mm lens (Nikon Micro-NIKKOR). The test tank was seeded with 20 $\upmu$m polyamide tracer particles (Orgasol 2002 D NAT 1; Arkema Group) to scatter the infrared illumination without affecting the copepods. The particles are nearly neutrally-buoyant (1.03 g $\hbox {cm}^{-3}$) and accurately move with the surrounding fluid.

Processing of the tomo-PIV images was performed using the DaVis 8.4 software package (LaVision GmbH). A calibration plate was traversed to six positions along the *z*-axis and provided calibration images for a preliminary mapping function. A self-calibration procedure corrected the mapping function for all cameras, thereby reducing calibration errors^29^.

The visual hull method was employed as a mask to eliminate the appearance of the *E. antarctica* specimen within the reconstructed volume^28,30^. Automating the image processing sequence to detect the silhouette of the *E. antarctica* specimen in the individual images streamlined the labor-intensive manual tracing used in prior studies^28^. A series of image filtering operations shown in Fig. 1b, including median and standard deviation filters, were applied to enhance the edge contrast, followed by applying the Canny edge detection method. Morphological operations, including dilation and erosion, were then used to refine the silhouette, effectively masking out noisy reconstructions near the organism’s body. This processing sequence was applied to the four simultaneous images, and a MLOS algorithm in DaVis 8.4 was employed to obtain a three-dimensional visual hull, which is shown in Fig. 1c.

Following the visual hull masking operation, particle intensity volumes were reconstructed using the MLOS-CSMART algorithm in DaVis 8.4, resulting in a measurement volume of -16 mm $<x<$ 16 mm, -7 mm $< y<$ 12 mm, and 0.5 mm $< z<$ 13.5 mm. The volume of velocity vectors was calculated by cross-correlating reconstructed volume pairs separated by $\Delta t = 5$ ms. The interrogation volume was 32 $\times$ 32 $\times$ 32 voxels, with a 75% overlap resulting in volumetric fields with a vector grid spacing of 0.21 mm. The velocity measurement uncertainty was estimated to be 0.7 mm $\hbox {s}^{-1}$.

*Euchaeta antarctica* kinematics were computed using the average of the three-dimensional locations of the points along the forepart of the head of the specimen (i.e., the rostrum) calculated from the visual hull analysis. From the tomo-PIV velocity measurements, the vorticity ($\omega$) was calculated using

1 $$\begin{aligned} \begin{bmatrix} \omega _x=\frac{1}{2}(\frac{du_z}{dy}-\frac{du_y}{dz}) \\ \omega _y=\frac{1}{2}(\frac{du_x}{dz}-\frac{du_z}{dx}) \\ \omega _z=\frac{1}{2}(\frac{du_y}{dx}-\frac{du_x}{dy}) \end{bmatrix} \end{aligned}$$

where $u_x$, $u_y$, and $u_z$ are the velocity components in the *x*, *y*, and *z* directions, respectively, and derivatives were calculated via central finite difference.

Previous research indicates that predators/prey interactions are mediated by velocity difference within the surrounding flow field^10,20,31^. Fluid strain rate, which can be separated into normal and shear components, quantifies the rate of deformation of material elements in the fluid motion. Notably, a copepod or other prey may not be aligned with the coordinate system when they detect fluid disturbances created by the predator (*E. antarctica* in this case), which presents a challenge in accurately quantifying the potential sensory cue created by the predator. To overcome this, the maximum strain rate ($E_{max}$) is utilized, which provides a coordinate-independent measure of hydrodynamic disturbances. This metric is calculated by extracting the eigenvalues from the strain rate tensor, with $E_{max}$ defined as the largest absolute value along the tensor’s principal axes^26^. This approach effectively decouples the orientation of the prey from the orientation of the *E. antartica* and the corresponding flow disturbance. The strain rate tensor (${\mathop {e}\limits ^\rightrightarrows }$) components were directly calculated using the measured volumetric velocity fields (in the coordinate system of the measurement volume):

2 $$\begin{aligned} \begin{bmatrix} e_{xx}=\frac{du_x}{dx} & e_{xy}=\frac{1}{2}(\frac{du_x}{dy}+\frac{du_y}{dx}) & e_{xz}=\frac{1}{2}(\frac{du_z}{dx}+\frac{du_x}{dz}) \\ e_{yx}=e_{xy} & e_{yy}=\frac{du_y}{dy} & e_{yz}=\frac{1}{2}(\frac{du_z}{dy}+\frac{du_y}{dz}) \\ e_{zx}=e_{xz} & e_{zy}=e_{yz} & e_{zz}=\frac{du_z}{dz} \end{bmatrix} \end{aligned}$$

and the eigenvalues, $\lambda$, were determined by

3 $$\begin{aligned} det({\mathop {e}\limits ^\rightrightarrows }-\lambda {\mathop {I}\limits ^\rightrightarrows })=0, \end{aligned}$$

where ${\mathop {I}\limits ^\rightrightarrows }$ is the identity matrix and *det* indicates the determinant operation for the tensor. This calculation yields three root values for $\lambda$ that correspond to the eigenvalues (i.e., $\lambda _1,\lambda _2,\lambda _3$) of the strain rate tensor. The maximum strain rate, then, is the maximum absolute value of the eigenvalues (i.e., the magnitude of the strain rate along the principal axes) of the strain rate tensor:

4 $$\begin{aligned} E_{max}=max(|\lambda _1|,|\lambda _2|,|\lambda _3|). \end{aligned}$$

The purpose and advantage of presenting the strain rate measurements in this framework is to eliminate the arbitrary orientation of the coordinate system, which is aligned with neither the copepod nor the potential prey or predator.

The dissipation rate of kinetic energy due to viscosity, $\Psi$, is also of interest because it provides a measure of the mechanical cost of propulsion through the viscous fluid. Further, a larger dissipation rate indicates that fluid velocity gradients are smoothed more rapidly, hence reducing the period that the flow disturbance may persist and be sensed in a predator/prey context. The viscous dissipation rate is another quantity calculated from the spatial gradients of the fluid velocity field:

5 $$\begin{aligned} \Psi =\mu \Biggl [2\Bigl [(\frac{\partial u_x}{\partial x})^2+(\frac{\partial u_y}{\partial y})^2+(\frac{\partial u_z}{\partial z})^2\Bigr ]+(\frac{\partial u_x}{\partial y}+\frac{\partial u_y}{\partial x})^2+(\frac{\partial u_x}{\partial z}+\frac{\partial u_z}{\partial x})^2+(\frac{\partial u_y}{\partial z}+\frac{\partial u_z}{\partial y})^2\Biggr ], \end{aligned}$$

where $\mu$ is the fluid dynamic viscosity. An additional advantage of the current tomo-PIV measurements is that each of these gradients may be calculated directly from the volumetric velocity field (again performed via central finite difference).

## Results

### Copepod kinematics

**Figure 2:**
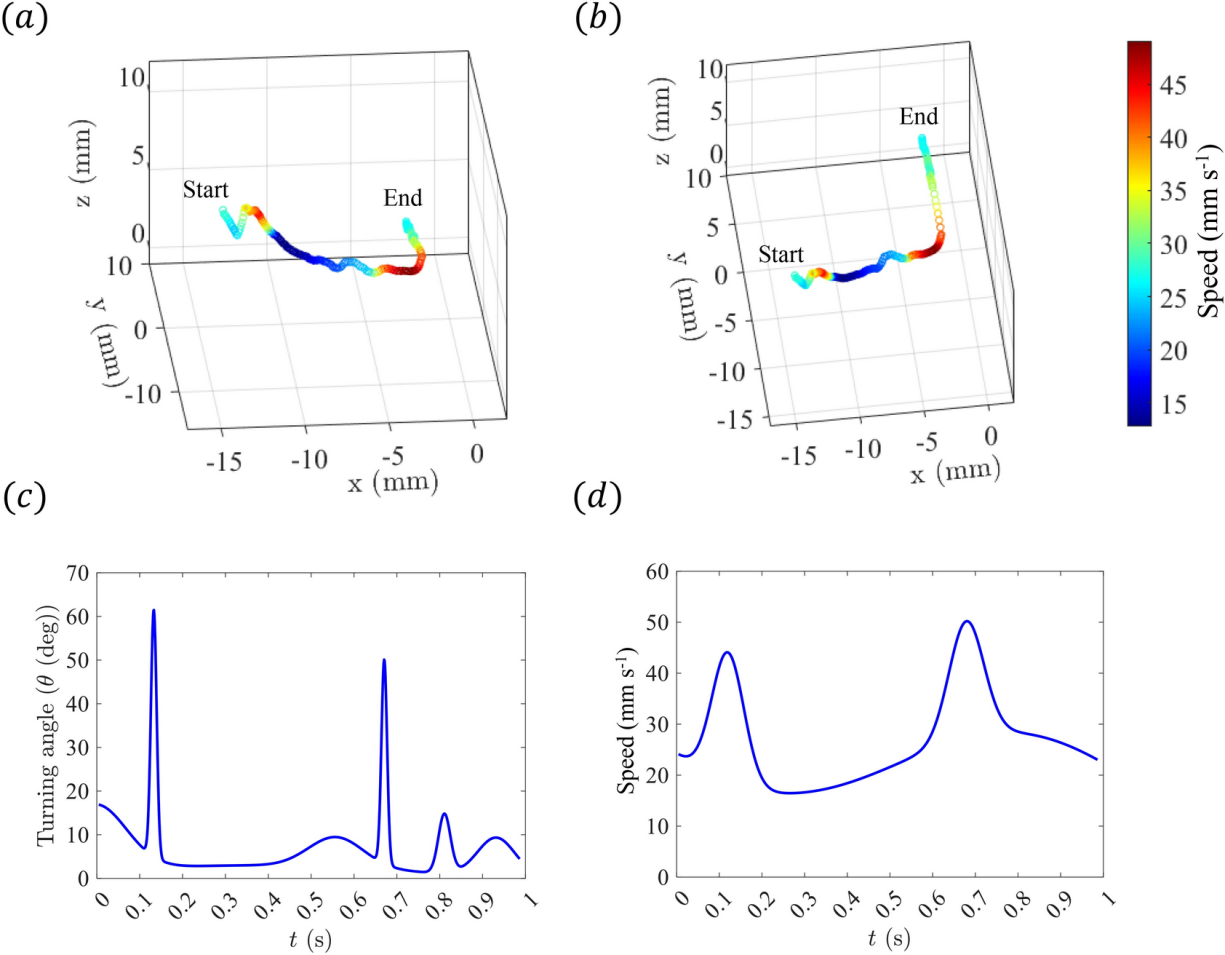
(**a,b**) Two perspectives of a 3D trajectory (of the forepart of the head; the rostrum) for the swimming *E. antarctica*. The local color of the trajectory indicates the instantaneous swimming speed. Corresponding time records of (**c**) turning angle and (**d**) swimming speed.

A three-dimensional trajectory of an adult *E. antarctica* during cruise swimming is depicted in Fig. 2a,b, with colors representing the swimming speed. This trajectory was selected as a typical example of the recorded swimming behavior of *E. antarctica* and additionally corresponded to a recording with excellent optical access around the specimen for subsequent tomo-PIV analysis of the surrounding fluid motion. The trajectory points specifically correspond to the forepart of the head (the rostrum). The trajectory covers a distance of roughly 25 mm and has a duration of roughly one second. This visualization captures the intricate movement patterns, emphasizing the organism’s ability to maneuver with a high degree of control in an aquatic environment. The trajectory includes two turns that are each followed by straight cruise swimming motions. Turning angle is defined as the angular change measured between the direction of two consecutive segments of the swimming trajectory. The time record of the turning angle (Fig. 2c) reveals two sharp peaks that correspond to rapid changes in the trajectory heading. The time record of the speed of the organism (Fig. 2d) varies in a manner that appears to be correlated with the turning angle. The copepod demonstrates elevated speed at roughly the same time points as the turning events, suggesting that the organism increases its propulsion thrust as it changes direction. The stroke frequency of the cephalic appendages that generate propulsive thrust varies in a manner that is consistent with these behaviors, as well. During the straight trajectory motion, the stroke frequency is 20 Hz, whereas during the higher-speed turning events, the stroke frequency is 34 Hz.

**Figure 3:**
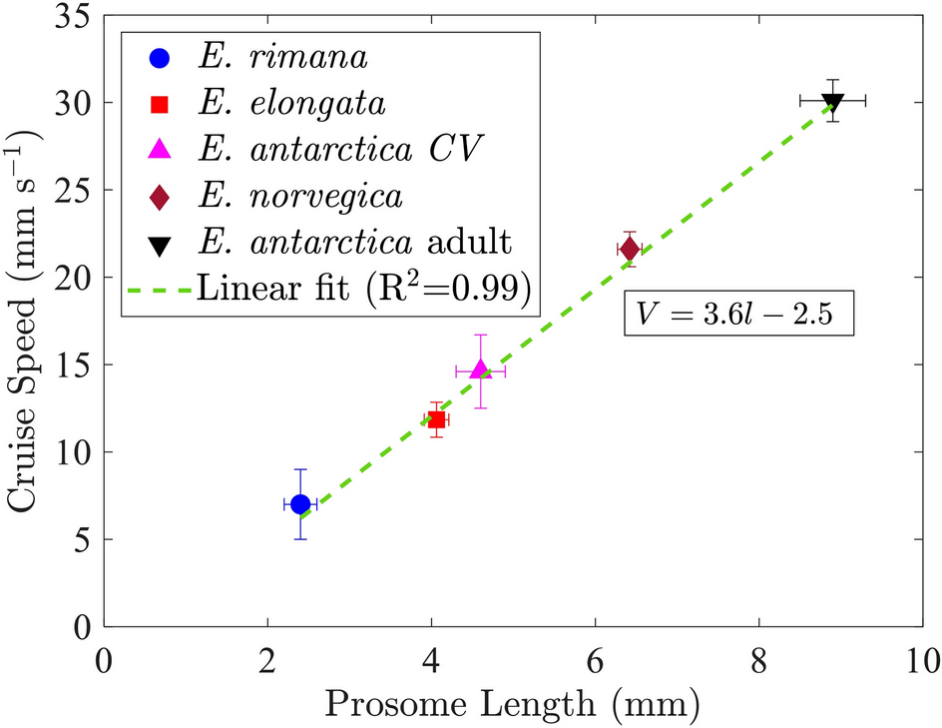
The relationship between the speed of free swimming *Euchaeta* and prosome length during cruise behavior. The error bars indicate the standard deviation of the data set. The number of individual samples from which the cruise velocity is computed: $N=42$ for *E. rimana*^22^, $N=8$ for *E. elongata*^26^, $N=38$ for *E. antarctica* CV^27^, $N=10$ for *E. norvegica* (K. Catton, unpublished data), and $N=9$ for *E. antarctica* adult (current data).

Figure 3 compares the swimming speed measured for nine adult *E. antarctica* specimens (during straight swimming) in this study to previous measurements of speed during cruise swimming for the genus *Euchaeta*. The plot shows a clear correlation between prosome length and swimming speed, indicating that larger specimens of the genus *Euchaeta* cruise faster than their smaller counterparts. The error bars associated with each data point correspond to the standard error in order to report the variation in length and speed of the specimens measured. The specimen for the presented flow field analysis in this study has a prosome length of 8.3 mm.

### Flow fields

**Figure 4:**
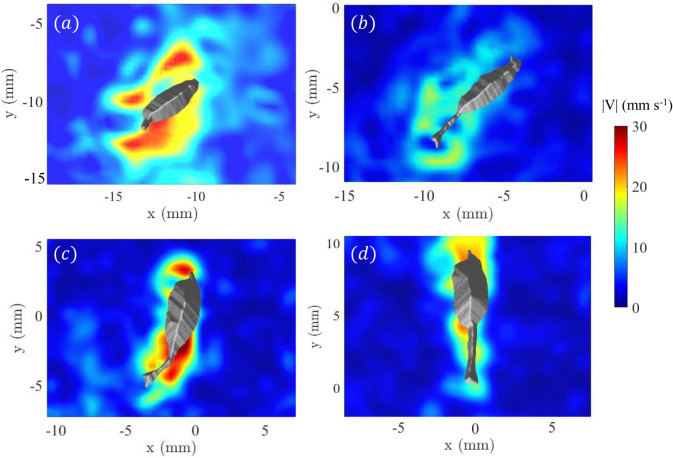
Fluid velocity magnitude on the mid-plane (relative to the animal body position in the *z*-direction) at (**a**) $t=0.1$ s (first turn/acceleration event), (**b**) $t=0.4$ s (during first straight motion period), (**c**) $t=0.68$ s (second turn/acceleration event), and (**d**) $t=1$ s (during second straight motion period). Figure generated with MATLAB.

Figure 4 presents the magnitude of the fluid velocity ($|V|=\sqrt{u_x^2+u_y^2+u_z^2}$) on the mid-plane (relative to the animal body position in the *z*-direction) of the swimming *E. antarctica* captured at four distinct time points: initial acceleration/turn, straight motion, second acceleration/turn, and second straight motion. The images reveal dynamic changes in the flow field as the organism maneuvers through the water. During the first turn and acceleration event (Fig. 4a), a high-velocity region is observed on each side of the organism in the $x-y$ plane, indicative of the powerful thrust generation required to accelerate and turn. During the straight cruising period (Fig. 4b), the fluid velocity around the organism is relatively smaller in magnitude and more spatially uniform, suggesting a balance between thrust and drag forces allowing for efficient sustained movement. This time point ($t = 0.4$ s) demonstrates the ability of *E. antarctica* to maintain a streamlined body position to minimize resistance and optimize forward propulsion. The next time point ($t = 0.68$ s) captures a second turn/acceleration event (Fig. 4c), in which the regions of large fluid velocity near the tail of the organism result from the significant tail and appendage movements involved in rapid directional changes. Finally, the second straight motion (Fig. 4d) is characterized by a significant decrease in the fluid velocity around and especially behind the organism, signifying a return to balanced thrust and drag forces after the turn.

**Figure 5:**
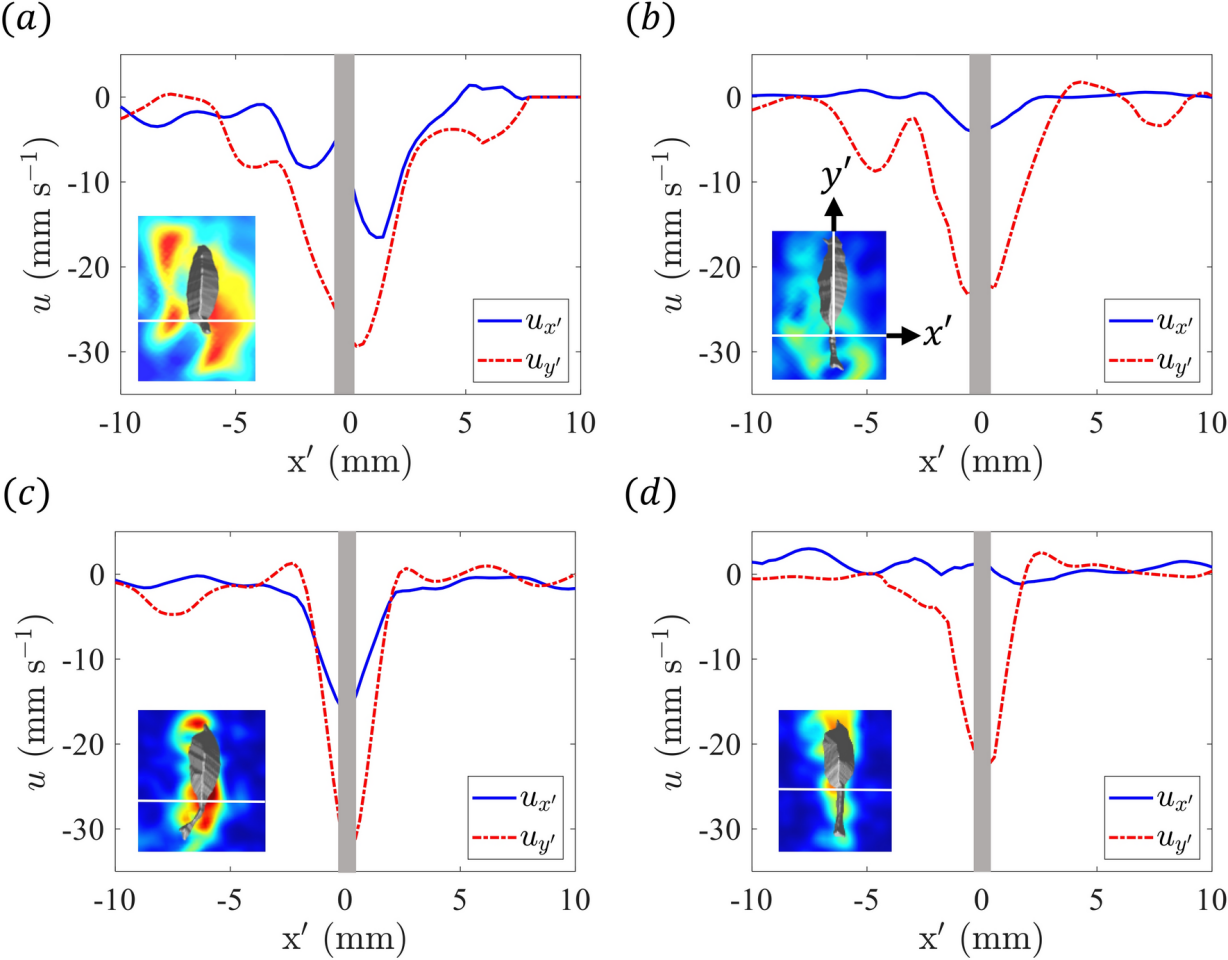
Transverse profiles of fluid velocity along a transect oriented at $90^\circ$ relative to the center axis of the organism and passing through the location of the base of the prosome (shown by the white line in the inset figures). The profiles correspond to $u_{x'}$ and $u_{y'}$ at (**a**) $t=0.1$ s (first turn/acceleration event), (**b**) $t=0.4$ s (during first straight motion period), (**c**) $t=0.68$ s (second turn/acceleration event), and (**d**) $t=1$ s (during second straight motion period). The gray region corresponds to the region where the animal body is located near $x'=0$. The inset figures show the fluid velocity magnitude on the mid-plane (relative to the animal body in the *z*-direction) in a rotated coordinate system that aligns $y'$ with the center axis of the organism. The datum for $x'$ is at the location of the copepod’s center axis. Figure generated with MATLAB.

Figure 5 reports transverse profiles of fluid velocity on the mid-plane (relative to the animal’s body position in the $z$-direction) at the same time points as Figure 4. The coordinate system ($x'-y'$) is rotated to align with the copepod’s central axis, allowing for examination of the flow patterns relative to the organism’s body orientation, as labeled in Fig. 5b. Two velocity profiles, $u_{x'}$ and $u_{y'}$, which report the fluid motion perpendicular and parallel to the copepod’s body axis, respectively, are shown. It should be noted that $u_{z'}$ is consistently near zero due to the alignment of the axes with the organism. Each profile in Fig. 5 corresponds to the $y'$ location at the base of the prosome.

The velocity profiles in Fig. 5a, c correspond to time points during the turn/acceleration motions and display elevated fluid velocity due to the organism’s active maneuvering. During each counter-clockwise turning motion (in the $x-y$ plane) at $t=0.1$ s (Fig. 5a) and $t=0.68$ s (Fig. 5c), $u_{x'}$ is negative close to the organism, indicating a strong flow toward the left in the inset field plots, due to the turning motion. This indicates leftward movement of the tail, consistent with the organism’s counter-clockwise turn. In contrast, during the straight copepod trajectory segments at $t=0.4$ s (Fig. 5b) and $t=1$ s (Fig. 5d), the $u_{x'}$ profiles are fairly flat and near zero, reflecting a more streamlined flow pattern during straight swimming motion. Additionally, the peak magnitude in the fluid velocity parallel to the copepod body axis, $u_{y'}$, is roughly 1.5 times larger during turning events compared to the straight motion periods (i.e., the peak velocity magnitude is near 30 mm $\hbox {s}^{-1}$ in Fig. 5a, c compared to near 20 mm $\hbox {s}^{-1}$ in Fig. 5b, d. This highlights the increased induced flow, which is connected to enhanced thrust force generation during the turn and acceleration events.

**Figure 6:**
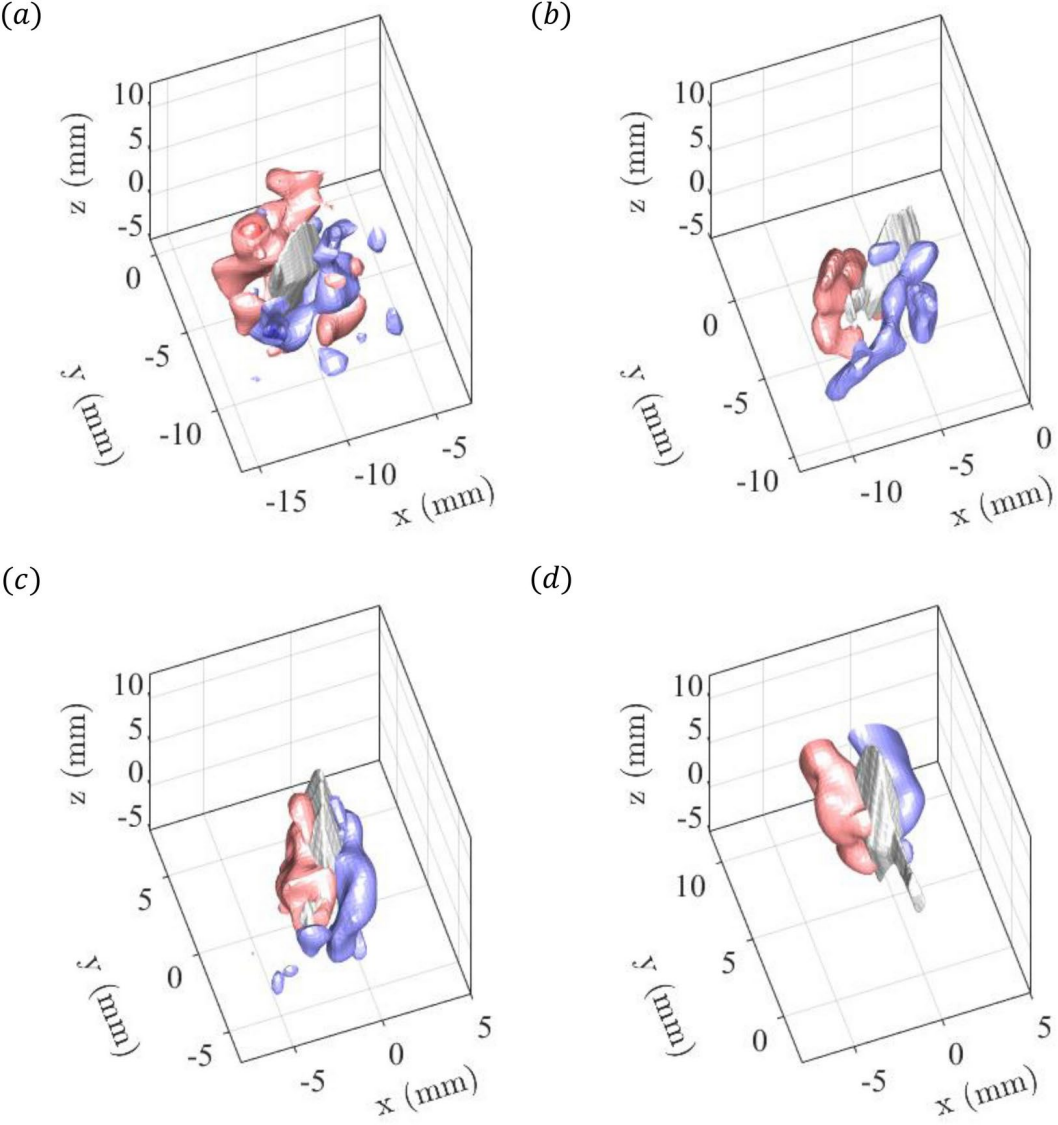
Iso-surfaces of regions of positive (red) and negative (blue) *z*-component of vorticity ($$\omega _z = \pm 15$$$$\hbox {s}^{-1}$$) at (**a**) $$t=0.1$$ s (first turn/acceleration event), (**b**) $$t=0.4$$ s (during first straight motion period), (**c**) $$t=0.68$$ s (second turn/acceleration event), and (**d**) $$t=1$$ s (during second straight motion period). Figure generated with MATLAB.

**Figure 7:**
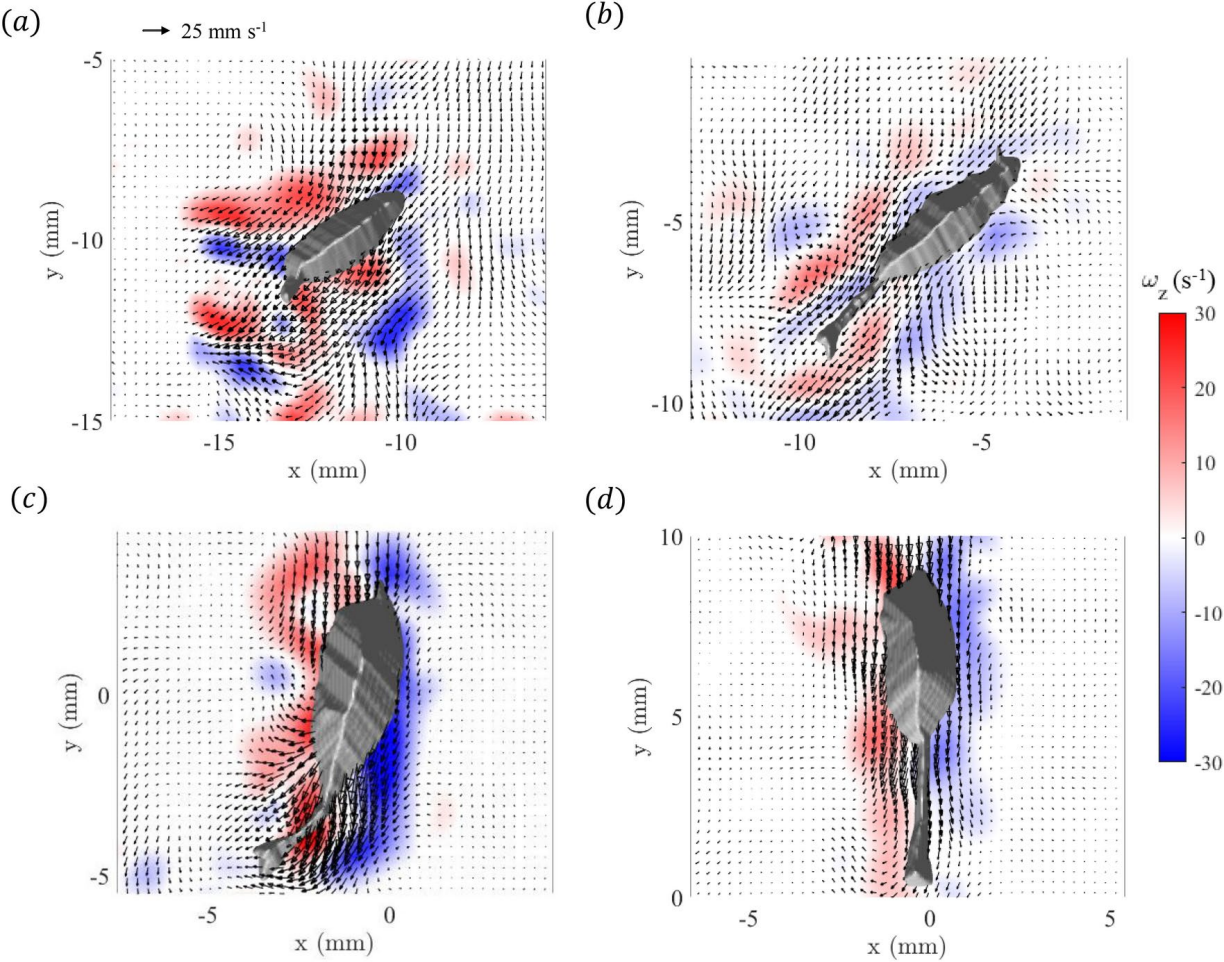
Vorticity (*z*-component) overlaid with velocity vectors on the mid-plane (relative to the animal body in the *z*-direction) at (**a**) $t=0.1$ s (first turn/acceleration event), (**b**) $t=0.4$ s (during first straight motion period), (**c**) $t=0.68$ s (second turn/acceleration event), and (**d**) $t=1$ s (during second straight motion period). Figure generated with MATLAB.

**Figure 8:**
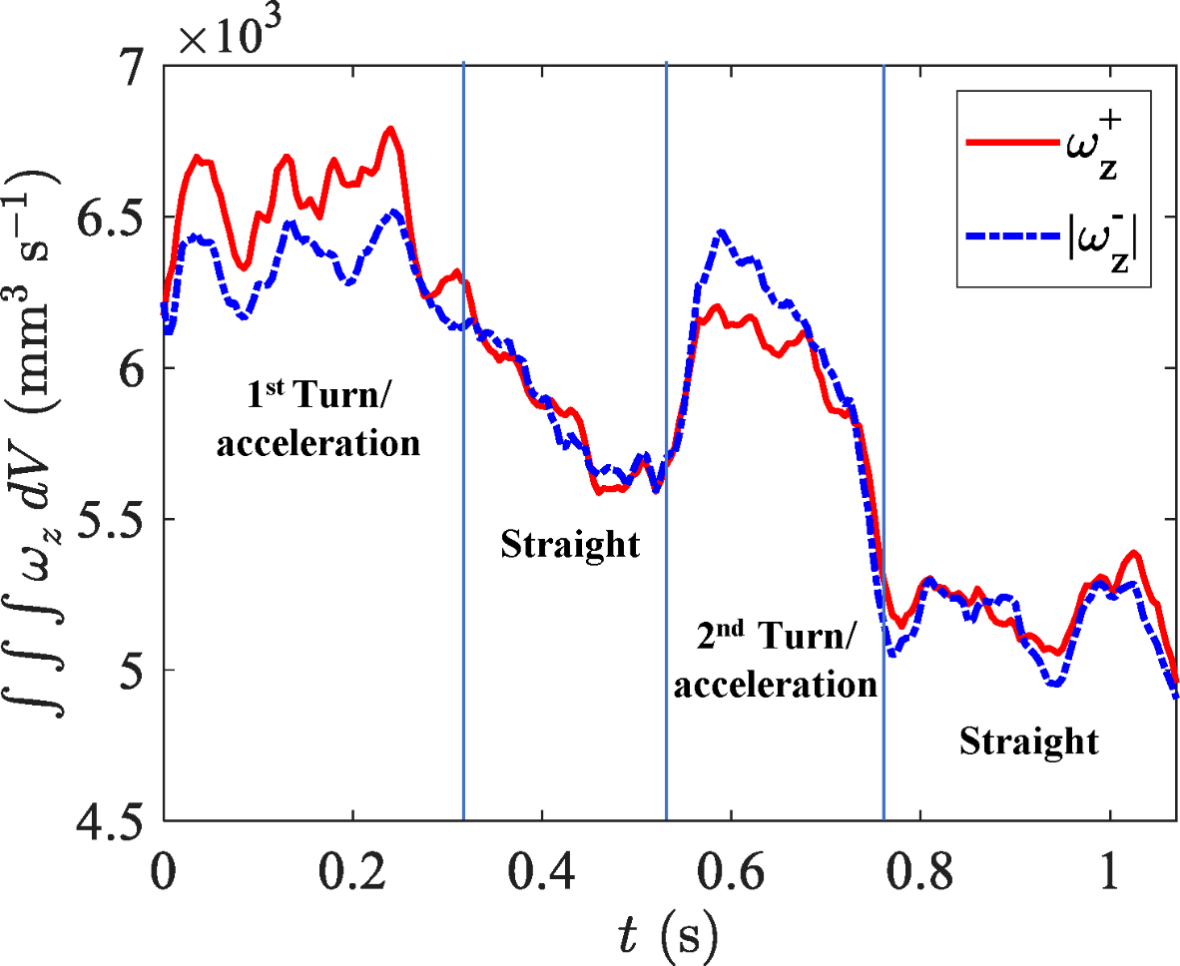
Time records of total vorticity (*z*-component) in the volume, integrated separately for positive values and (the magnitude of) negative values.

The fluid vorticity (*z*-component) around swimming *E. antarctica* is depicted and analyzed in Figs. 6, 7, and 8. Given that the animal exhibits two counter-clockwise turns in the $x$-$y$ plane, the $z$-component of vorticity ($\omega _z$) is particularly relevant to examine the shear layers along the side of the copepod. Figure 6 presents iso-surfaces of positive (red) and negative (blue) regions of the *z*-component of vorticity, highlighting the generation and evolution of vorticity as the copepod maneuvers through the water. At the time point of the first turn/acceleration event (Fig. 6a), the iso-surfaces reveal volumes of elevated vorticity that are convoluted and disordered in spatial arrangement. The volumes of elevated vorticity are more coherent and organized during the second turn/acceleration event (Fig. 6c), but the volumes of elevated vorticity are large (consistent with Fig. 6a) due to the strong shear layers along the side of copepod. During the straight trajectory periods (Fig. 6b, d), coherent volumes of positive and negative vorticity appear along the sides of the copepod due to the flow shear on the left and right sides of the organism.

Figure 7 shows the *z*-component of vorticity overlaid with velocity vectors on the midplane (relative to the animal body position in the *z*-direction). During the turn/acceleration events, the distribution of vorticity in the midplane is marked by intense regions of both positive and negative vorticity, reflecting the dynamic changes in the fluid flow as the copepod redirects its path (Fig. 7a, c). In contrast, the straight swimming phases exhibit weaker regions of vorticity on either side of the copepod (Fig. 7b, d). In these cases, the velocity vector field reveals a streamlined flow pattern along the sides of the organism, which are highlighted by the relatively modest vorticity regions in the shear layers. The peak values of vorticity during the turn/acceleration events are around 30 $\hbox {s}^{-1}$ and -25 $\hbox {s}^{-1}$, which is approximately 1.67 to 2 times larger than the peak values during the straight motions at around ± 15 $\hbox {s}^{-1}$.

Figure 8 provides a temporal record of the magnitude of the total (i.e., volume integrated) values of positive and negative *z*-component of vorticity. The time records provide an integrated view of the copepod’s influence on its fluid environment at each time point in the trajectory. The time records for positive and negative vorticity are near mirrors of each other, with peaks and troughs of the absolute value occurring during the same time segments. The total vorticity exhibits two peaks corresponding to the turns with subsequent troughs during straight swimming, reflecting the alternating dynamics between high-energy maneuvers during turn/acceleration events and less energetic motions during straight cruise swimming.

**Figure 9:**
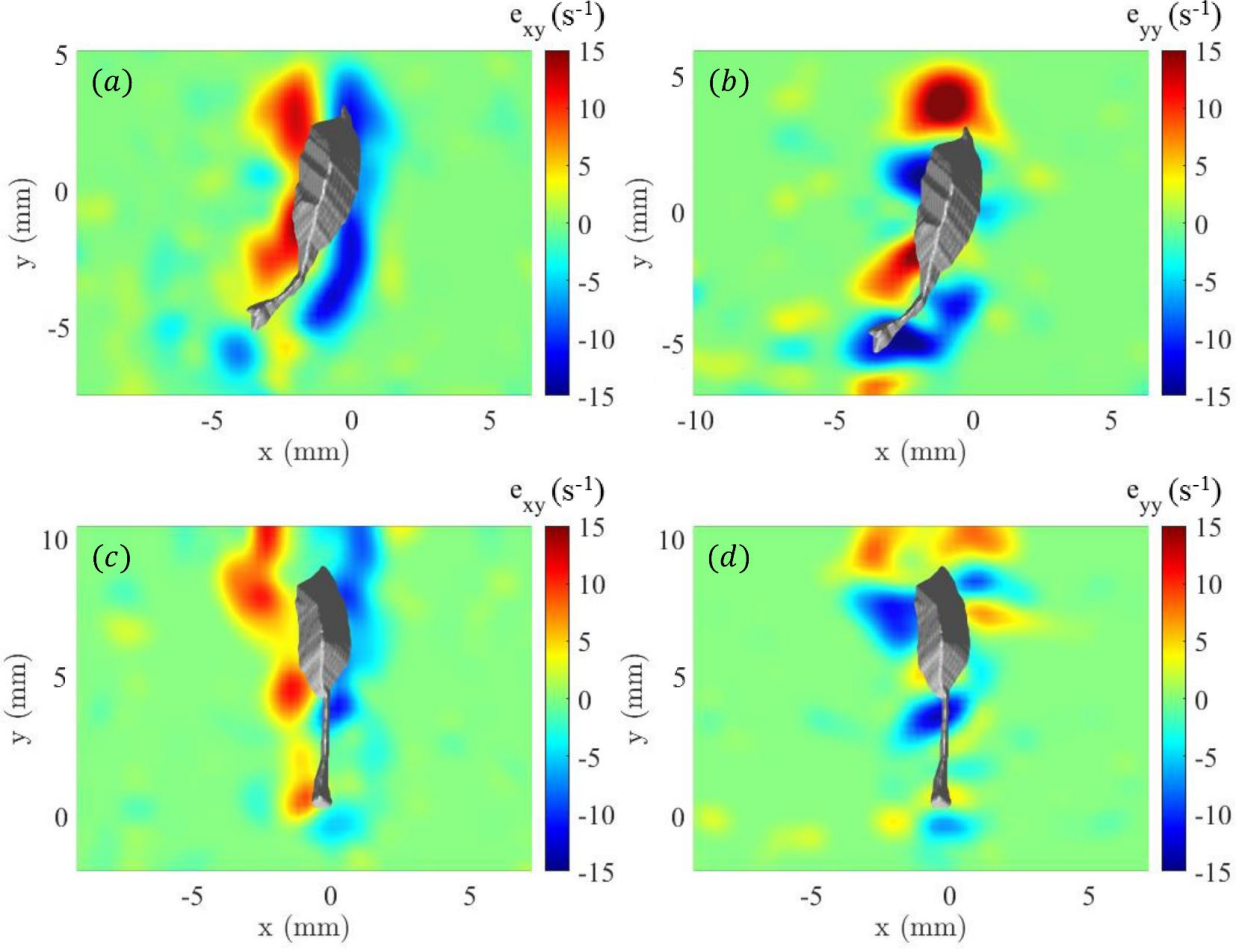
Fluid strain rate components on the mid-plane (relative to the animal body in the *z*-direction) for (**a,c**) shear strain rate, $e_{xy}$, and (**b,d**) normal strain rate, $e_{yy}$, at (**a,b**) $t=0.68$ s (second turn/acceleration event), and (**c,d**) $t=1$ s (during second straight motion period). Figure generated with MATLAB.

Figure 9 provides a detailed view of the largest components of the fluid strain rate tensor ($e_{xy}$ and $e_{yy}$) on the midplane (relative to the animal body position in the *z*-direction) at two time points. Figure 9a, c display the shear strain rate component ($e_{xy}$) during the second turn/acceleration event and a straight swimming motion, respectively. At $t=0.68$ s (Fig. 9a), elevated regions of shear strain rate adjacent to the organism are observed. These elevated levels of $e_{xy}$ correspond with the active turning maneuver and suggest significant fluid deformation due to the copepod’s rapid directional change and increase in swimming speed. In the subsequent straight swimming phase at $t=1$ s (Fig. 9c), the shear strain rate appears relatively less intense around the copepod. Figure 9b, d show the normal strain rate component ($e_{yy}$), which can be associated with extension or compression in the flow. During the second turn/acceleration event at $t=0.68$ s (Fig. 9b), there is a clear asymmetry in intense regions of $e_{yy}$ on opposite sides of the copepod body that suggest the flow is being compressed on one side and extended on the other. At $t=1$ s (Fig. 9d), the normal strain rate is relatively smaller in magnitude, consistent with a streamlined flow pattern that would be expected when the copepod is swimming in a straight trajectory path.

## Discussion

The present study, with its unique approach, quantitatively assesses the hydrodynamic disturbances generated by adult *E. antarctica* during cruise swimming in straight and turning motions. This study offers fresh and intriguing insights into the locomotion and ecological interactions of *E. antarctica* in the Southern Ocean. In broad terms, the current volumetric tomo-PIV data confirm an intricate interaction between the adult *E. antarctica* and the surrounding fluid motion during cruise swimming, which is consistent with observations of other copepod species^18,26,27,32^.

### Cruise speed and kinematics

The linear regression between cruise speed and prosome length (shown in Fig. 3) reveals a seemingly simple relationship. The relationship suggests that cruise swimming in *Euchaeta* follows a size-proportionality trend and that cruise velocity in *Euchaeta* can be reliably estimated from prosome length. Drag forces typically scale on area, i.e., projected area for form drag and surface area for shear drag, hence implying an expected non-linear relationship with prosome length. Indeed, Svetlichny et al.^7^ found that over a wide range of copepod species, the cruise speed scales with prosome length to the power of 1.4 (for copepods with prosome length less than 4 mm). In the case of *Euchaeta* presented here, the complex interaction of size, temperature, and fluid viscosity combined with similar morphology across species size leads to the observed linear relationship. Based on the current recordings, *E. antarctica* increases the beat frequency of its cephalic appendages to increase its cruise swimming speed. Appendage beat frequency data are unavailable for the smaller *Euchaeta* species for comparison, but the current data agree well with the trend reported by Svetlichny et al.^7^ over a wide range of copepod species for frequency as a function of prosome length (again noting that their data are for prosome length less than 4 mm, which is much smaller than *E. antarctica*).

Water temperature also varies across prosome length for the *Euchaeta* genus since the species size increases with latitude. Fluid viscosity increases as the temperature decreases; hence, the larger *E. antarctica* specimens are moving through an enhanced viscous environment (i.e., kinematic viscosity is $$\nu = 1.8$$$$\hbox {mm}^2$$$$\hbox {s}^{-1}$$ for high latitude seawater at $$0^\circ$$C, whereas $$\nu = 1.0$$$$\hbox {mm}^2$$$$\hbox {s}^{-1}$$ for tropical latitudes at $$23^\circ$$C). Despite the variation in viscosity, the size-proportional trend during cruise swimming dominates the balance of inertial effects compared to viscous effects, as quantified by the Reynolds number (*Re*). Since the length and velocity scales appear in the numerator of the *Re* formulation (i.e., $$Re=UL/\nu$$), *Re* is larger for the larger species that are moving faster. The fluid viscosity varies inversely with temperature, but the changes are relatively modest compared to the changes in length and velocity. *Re* for the adult *E. antarctica* reported here is roughly 145. For comparison, the $$Re =17$$ value for *E. rimana*, at the smaller end of the size range, is roughly an order of magnitude smaller. Hence, *Euchaeta* clearly does not follow dynamic similarity (i.e., maintaining constant *Re*) across congeners. Nevertheless, each species swims in an intermediate *Re* number range in which inertial and viscous effects each play an influencing role, leading to a compromise of the effects of body size, fluid viscosity, and swimming speed. The linear relationship of swimming speed and prosome length also suggests a potential adaptation to the thermal conditions of their environment, where larger organisms within this genus may have enhanced appendage stroking efficiency or greater muscle power to achieve greater speeds. Further, larger body sizes and faster cruising speeds may confer advantages in colder habitats, such as enhanced metabolic efficiency or improved predation^26^.

### Flow disturbance

The detailed velocity measurements show a strong flow disturbance surrounding the cruising adult *E. antarctica*. The fluid velocity is elevated in the region adjacent to the organism, with the elevated fluid velocity region extending 2-4 mm from the copepod body. The observed fluid velocity peaks at approximately 21-23 mm $\hbox {s}^{-1}$ during straight cruising - a sharp increase from the 12 mm $\hbox {s}^{-1}$ recorded for *E. antarctica* CV^27^. The peak fluid velocity is also smaller for the smaller species *E. elongata* (10 mm $\hbox {s}^{-1}$) and *E. rimana* (7.5 mm $\hbox {s}^{-1}$), as reported by Catton et al.^26^. Clearly, the larger fluid velocity corresponds to the elevated swimming speed for the larger *E. antarctica* adult.

The velocity decreases rapidly with distance from the copepod, creating an intense shear layer (Fig. 5). The enhanced vorticity regions observed along the sides of the copepod correspond to the shear layers (as seen in Figs. 6 and 7). A similar pattern of vorticity distribution was quantified in the 2D measurements for *E. antarctica* CV in Catton et al.^27^, with patches of opposite sign vorticity located along the sides of the copepod. The magnitude of the peak value of vorticity is similar, with roughly 20 $\hbox {s}^{-1}$ for *E. antarctica* CV^27^, 15 $\hbox {s}^{-1}$ for straight swimming, and 25-30 $\hbox {s}^{-1}$ during turn/acceleration events for the adult. It is also clear that the spatial extent of the regions of elevated vorticity is much larger for the adult specimen compared to the CV, which, of course, relates to their relative body sizes. As noted above, cruise speed and fluid velocity are larger for the adult. Hence, the combination of a larger change in fluid velocity across the shear layer occurring over a greater spatial distance (due to the size-proportional trend) yields similar velocity gradient magnitudes and, hence, similar vorticity values in the shear layers.

The detailed fluid velocity measurements reveal a remarkable contrast between straight cruising motion and turn/acceleration events. The two prominent peaks observed in the time record of turning angle correspond to major directional changes. These events suggest that *E. antarctica* can quickly alter its course, potentially as a predator evasion strategy or while pursuing prey. The copepod performed the turn/acceleration events at elevated swimming speed (Fig. 3). Consistently, the copepod increased the beat frequency of its cephalic appendages to achieve elevated swimming speed. The peak fluid velocity near the organism correspondingly increases to 31-33 mm $\hbox {s}^{-1}$ during the turn/acceleration events, which is approximately 1.5 times greater than the fluid velocity during straight swimming.

During periods of straight cruise swimming, the fluid motion follows a streamlined pattern in the dorso-ventral view (most clearly seen in Fig. 7 (*b*) and (*d*)), which is familiar based on lower-resolution 2D measurements surrounding other copepods^15,26,27,33^. However, during turns, the flow field undergoes a significant, tail-induced distortion where the location of maximum fluid velocity shifts from the appendage region to near the tail (most clearly seen in Fig. 4 (*a*) and (*c*)). The shear layer intensity also increases during the turn/acceleration events, which is most clearly identified in the time records of the volume-integrated vorticity (z-component) where the magnitude in each shear layer is elevated during the turn/acceleration events (Fig. 8). These time records reveal symmetry between the left and right shear layers during straight motion, but a small imbalance between positive and negative vorticity was observed during turns. Such rapid turn maneuvers reveal the ability for swift directional changes, which may serve multiple ecological functions, including re-positioning relative to environmental flow structure, increasing encounter rates with prey, or minimizing exposure to predators. The difference in total z-component of vorticity between the two periods of straight cruise swimming can be attributed to the residual vorticity generated during the preceding turning maneuvers. The first turn produces stronger vorticity structures, which persist into the first straight segment, resulting in higher total vorticity. By contrast, the second turning event induces weaker vorticity, and therefore the vorticity decreases more rapidly during the second straight motion, leading to lower overall vorticity levels.

The peak values and asymmetry in vorticity during turns in *E. antarctica* are similarly observed in turns of other aquatic organisms. Studies have shown that fish (at much larger *Re*) generate complex wake patterns with enhanced vorticity during turns compared to swimming in a straight motion, which is attributed to the lateral forces exerted by their bodies and fins to create stronger vortical structures for rapid directional changes^34–36^. Moreover, the slight imbalance between positive and negative vorticity observed along the shear layers of *E. antarctica* during turns, compared to the balanced vorticity pattern during straight swimming, reflects the use of body movement to facilitate swift directional changes (Fig. 8). This concept was also observed by Dabiri et al.^37^, who reported that jellyfish and zebrafish create asymmetrical vorticity regions and pressure gradients as a result of their body motion to assist in turning.

For the adult *E. antarctica*, the peak value of viscous dissipation rate observed is 52 W $\hbox {m}^{-3}$ during the straight swimming period and 76 W $\hbox {m}^{-3}$ during the turn/acceleration events, which is roughly twice the peak calculated by Catton et al.^27^ for the *E. antarctica* CV (28-30 W $\hbox {m}^{-3}$, based on estimates from 2D data). The dissipation rate field may be integrated over the fluid volume to determine the total rate of energy dissipated in the flow disturbance. During straight swimming periods, the current data yield a total dissipation rate of 2.6-2.8${\times }10^{-8}$ W. Resulting from the enhanced swimming speed and elevated fluid velocity during the turn/acceleration events, the total dissipation rate increases to 3.5-3.8${\times }10^{-8}$ W. Despite not having full volumetric data (which required estimates for both the velocity gradient calculations and the volume integration step), Catton et al.^27^ reported the total dissipation for the *E. antarctica* CV as 1.0${\times }10^{-8}$ W. It is fascinating that the total dissipation rates differ by only a factor of 2.6 to 3.8 between the adult and CV *E. antarctica* despite the large differences in swimming speed and size (which potentially influences volumetric quantities as a cubed function). As related to the discussion above for the vorticity field, the compensation of the velocity gradients is part of the explanation (i.e., a larger velocity difference over a larger distance yields a similar gradient). There is also a substantial difference in the volume of the integration, which likely contributes to the larger total dissipation rate for the adult specimen. As another comparison point, Yen et al.^15^ reported a total dissipation rate for *E. rimana* of 9.3${\times }10^{-10}$ W. While this value is more than an order of magnitude smaller than the values reported above, there are significant caveats that the copepod specimen was described in the study as “stationary”, and their particle tracking data were very low resolution and also necessitated assumptions and estimates to calculate the total dissipation rate from the 2D field.

### Hydrodynamical signaling

The fluid strain rate is an important signal for prey to avoid predators, such as *Euchaeta*, as prey will respond with an escape^20,21^. As described above, the flow disturbance generated by the adult *E. antarctica* is considerable in strength and spatial extent. The shear strain rate ($e_{xy}$) reveals a similar spatial pattern in the shear layers as previously discussed for vorticity. Specifically, regions of different-signed elevated shear strain rate appear along the sides of the copepod in the dorsal-ventral view (Fig. 9 (*a*) and (*c*)). Further, the normal strain rate component during the turn/acceleration event reveals an intense asymmetric spatial pattern with compression and extension on the opposite sides of the copepod (Fig. 9 (*b*)), which could influence the copepod’s perception of its three-dimensional environment, affecting how it navigates and responds to external stimuli.

For each strain rate component, the strength is greater during the turn/acceleration events (peak of 21 $\hbox {s}^{-1}$) compared to the periods of straight swimming motion (peak of 11 $\hbox {s}^{-1}$) in which the copepod appears to minimize the flow disturbance, likely to maintain energy efficiency and perhaps to reduce hydrodynamic signals that could alert predators or prey. The elevated intensity during the turn/acceleration events is consistent with other flow characteristics discussed above. As a comparison point, Catton et al.^27^ reported a peak value for the normal strain rate component of 10 $\hbox {s}^{-1}$ for *E. antarctica* CV, which is remarkably consistent in magnitude with the results for straight cruising reported in the current data. Although not reported explicitly in the paper, the data in Catton et al.^26^ yield a peak for $E_{max}$ of 9 $\hbox {s}^{-1}$ for *E. elongata* and 10 $\hbox {s}^{-1}$ for *E. rimana*, which is also remarkably consistent with the adult (in straight cruising) and CV *E. antarctica*. Again, the relatively modest differences in the peak strain rate values, despite the technical limitations of previous data, are explained by the velocity gradients remaining relatively constant due to the specimens following a size-proportional trend, as discussed above.

**Figure 10:**
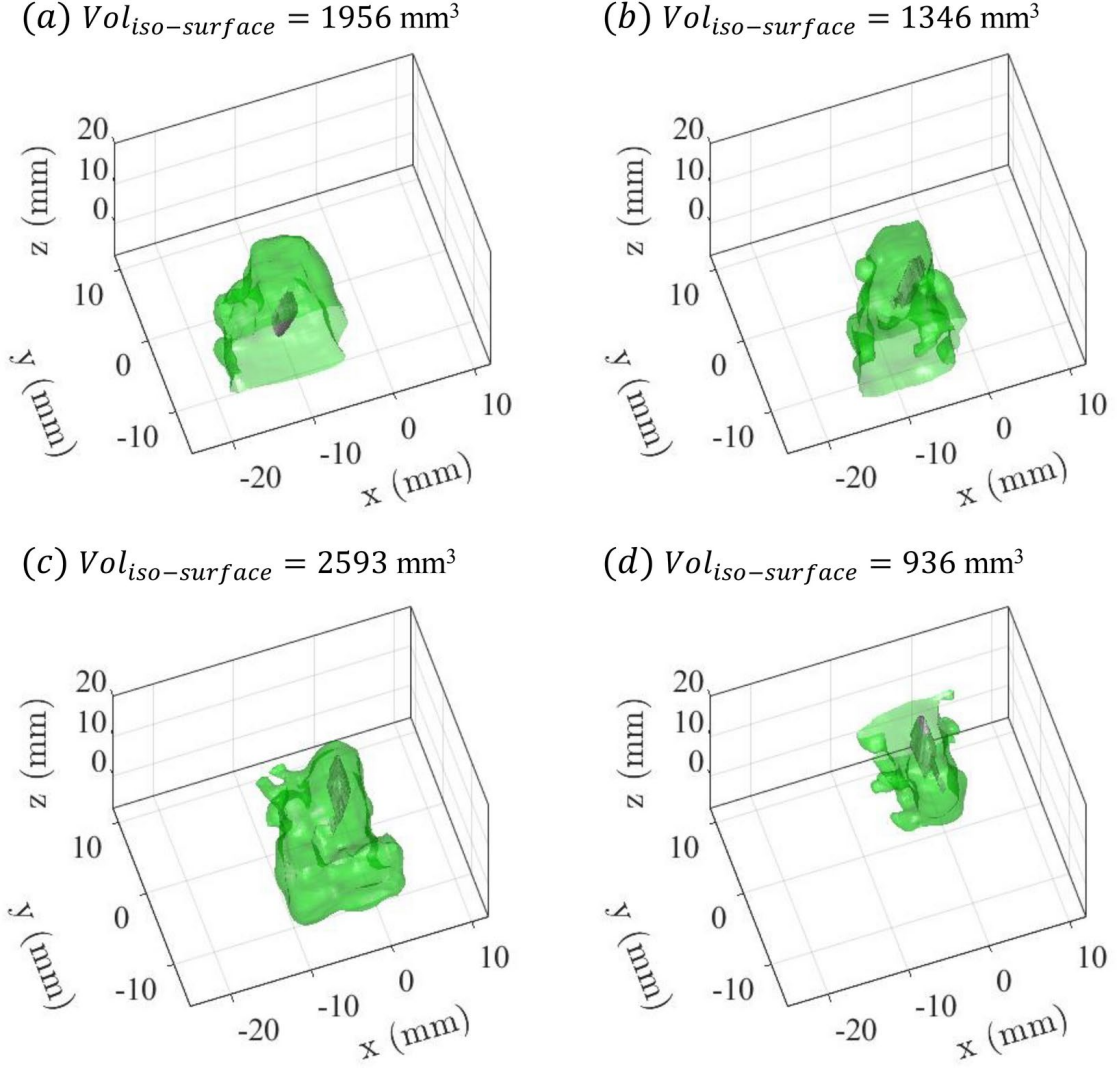
The 0.5 $\hbox {s}^{-1}$ iso-surface of the maximum deformation rate ($E_{max}$) at (**a**) $t=0.1$ s (first turn/acceleration event), (**b**) $t=0.4$ s (during first straight motion period), (**c**) $t=0.68$ s (second turn/acceleration event), and (**d**) $t=1$ s (during second straight motion period). $E_{max}$ of 0.5 $\hbox {s}^{-1}$ provides a representative value that has been observed to induce escape response in copepods (e.g.,^10,21,31^). Figure generated with MATLAB.

As noted by Catton et al.^27^, the volume surrounding a predatory copepod that exceeds a critical strain rate threshold is much larger than the copepod exoskeleton, hence increasing the predator’s conspicuousness to potential prey (that are sensitive to hydrodynamic cues). Catton et al.^27^ employed the 0.5 $$\hbox {s}^{-1}$$ contour for strain rate to represent a typical threshold to induce an escape. Based on planar PIV measurements, the 0.5 $$\hbox {s}^{-1}$$ contour area in the dorso-ventral view extends to 11 times the exoskeletal form of the *E. antarctica* CV. The current data facilitate consideration of the total volume of the flow disturbance and potential sensory cue. Figure 10 presents the iso-surface for the same sensory threshold level of $$E_{max} = 0.5$$$$\hbox {s}^{-1}$$. The size of the iso-surface highlights the extensive region surrounding the copepod that may present a hydrodynamic cue to potential prey. The figure panels report the total volume enclosed by the iso-surface. Note that these estimates may be slightly under-valued since the iso-surface extends to the measurement domain boundary, hence clipping the volume calculation. The visual hull volume may be used as a surrogate for the volume of the copepod exoskeleton. Hence, the volume enclosed by the $$E_{max} = 0.5$$$$\hbox {s}^{-1}$$ iso-contour is 11-13 times the copepod exoskeleton volume during the straight swimming motion and 22-25 times during the turn/acceleration events. It is impractical to compare the planar measurements described above quantitatively, but each set of measurements confirms the large spatial extent of the hydrodynamic cue.

### Conclusion

This study comprehensively analyzes the hydrodynamic characteristics surrounding adult *E. antarctica* during straight cruise swimming and turn/acceleration motions utilizing the tomo-PIV technique. The comparative analysis between straight swimming and turn/acceleration motions in adult *E. antarctica* demonstrates an ability to alter its course rapidly. During straight swimming, the fluid velocity field is characterized by a streamlined flow pattern with fluid shear layers on each side of the copepod for efficiency beneficial for sustained propulsion and energy conservation. In contrast, turn/acceleration maneuvers exhibit a dramatic increase in the complexity of the fluid velocity and vorticity fields, indicative of the rapid, directional change. The turn/acceleration events also demonstrate a period of heightened viscous dissipation rate, thus revealing an added cost of propulsion to generate the higher-speed turns. These maneuvers generate significant hydrodynamic disturbances; notably, a sharp increase in strain rate during the turn/acceleration events suggests increased conspicuousness during rapid turns. This contrast in hydrodynamic cue signatures between straight cruising and turn/acceleration maneuvering highlights the dual demands of population efficiency and survival agility faced by *E. antarctica* in the challenging Southern Ocean ecosystem. Such insights reveal the intricate interactions between morphology, behavior, and the environment.

The study also provides a comparative perspective across different species of the *Euchaeta* genus. Larger species like *E. antarctica* exhibit larger cruising speeds compared to their smaller counterparts such as *E. rimana* and *E. elongata*, reflecting a size-proportional trend in organism swimming speed and flow velocity. Despite variations in body size and environmental temperature, the fluid velocity gradient quantities, such as vorticity and shear strain rate, remain relatively consistent across species. This consistency is connected to the linear relationship found between prosome length and swimming speeds among different *Euchaeta* species during cruising, and the size-proportional trend ultimately dictates effective propulsion and sensory interactions across different ecological niches.

## Data Availability

The data used and analyzed during the current study will be provided from the corresponding author upon reasonable request.

## References

[1] Hopkins, T. The zooplankton community of Croker passage, Antarctic Peninsula. *Polar Biol. ***4**, 161–170. 10.1007/BF00263879 (1985).

[2] Żmijewska, M. & Yen, J. Seasonal and diel changes in the abundance and vertical distribution of the Antarctic copepod species *Calanoides acutus*, *Calanus propinquus*, *Rhincalanus gigas*, *Metridia gerlachei* and *Euchaeta antarctica* (Calanoida) in Croker Passage (Antarctic Peninsula). *Oceanologia. ***35**, 101–127 (1993).

[3] Yen, J. Predatory feeding behavior of an Antarctic marine copepod, *Euchaeta antarctica*. *Polar Res. ***10**, 433–442. 10.1111/j.1751-8369.1991.tb00664.x (1991).

[4] Greene, C. Foraging tactics and prey-selection patterns of omnivorous and carnivorous calanoid copepods. *Hydrobiologia. ***167**(168), 295–301. 10.1007/BF00026317 (1988).

[5] Jiang, H., Osborn, T. & Meneveau, C. The flow field around a freely swimming copepod in steady motion. Part I: Theoretical analysis. *J. Plankton Res. ***24**, 167–189. 10.1093/plankt/24.3.167 (2002).

[6] Kiørboe, T., Jiang, H. & Colin, S. Danger of zooplankton feeding: The fluid signal generated by ambush-feeding copepods. *Proc. R. Soc. B. ***277**, 3229–3237. 10.1098/rspb.2010.0629 (2010). 20538648 10.1098/rspb.2010.0629 PMC2981922

[7] Svetlichny, L., Larsen, P. & Kiørboe, T. Kinematic and dynamic scaling of copepod swimming. *Fluids. ***5**, 68. 10.3390/fluids5020068 (2020).

[8] Strickler, J. Calanoid copepods, feeding currents, and the role of gravity. *Science. ***218**, 158–160. 10.1126/science.218.4568.158 (1982). 17753444 10.1126/science.218.4568.158

[9] van Duren, L. & Videler, J. Escape from viscosity: The kinematics and hydrodynamics of copepod foraging and escape swimming. *J. Exp. Biol. ***206**, 269–279. 10.1242/jeb.00079 (2003). 12477897 10.1242/jeb.00079

[10] Fields, D. & Yen, J. The escape behavior of marine copepods in response to a quantifiable fluid mechanical disturbance. *J. Plankton Res. ***19**, 1289–1304. 10.1093/plankt/19.9.1289 (1997).

[11] Doall, M., Strickler, J., Fields, D. & Yen, J. Mapping the free-swimming attack volume of a planktonic copepod,* Euchaeta rimana*. *Mar. Biol. ***140**, 871–879. 10.1007/s00227-001-0735-z (2002).

[12] Kiørboe, T., Andersen, A., Langlois, V., Jakobsen, H. & Bohr, T. Mechanisms and feasibility of prey capture in ambush-feeding zooplankton. *Proc. Natl. Acad. Sci. ***106**, 12394–12399. 10.1073/pnas.0903350106 (2009). 19622725 10.1073/pnas.0903350106 PMC2718367

[13] Jiang, H. & Kiørboe, T. Propulsion efficiency and imposed flow fields of a copepod jump. *J. Exp. Biol. ***214**, 476–486. 10.1242/jeb.049288 (2011). 21228207 10.1242/jeb.049288

[14] Murphy, D., Webster, D. & Yen, J. A high-speed tomographic PIV system for measuring zooplanktonic flow. *Limnol. Oceanogr. Methods. ***10**, 1096–1112. 10.4319/lom.2012.10.1096 (2012).

[15] Yen, J., Sanderson, B., Strickler, J. & Okubo, A. Feeding currents and energy dissipation by *Euchaeta rimana*, a subtropical pelagic copepod. *Limnol. Oceanogr. ***36**, 362–369. 10.4319/lo.1991.36.2.0362 (1991).

[16] Yen, J. & Strickler, J. Advertisement and concealment in the plankton: What makes a copepod hydrodynamically conspicuous?. *Invertebr. Biol. ***115**, 191–205. 10.2307/3226930 (1996).

[17] Fields, D. & Yen, J. Implications of the feeding current structure of *Euchaeta rimana*, a carnivorous pelagic copepod, on the spatial orientation of their prey. *J. Plankton Res. ***19**, 79–95. 10.1093/plankt/19.1.79 (1997).

[18] Tiselius, P., Jonsson, P., Kaartvedt, S., Olsen, E. & Jørstad, T. Effects of copepod foraging behavior on predation risk: An experimental study of the predatory copepod *Pareuchaeta norvegica* feeding on *Acartia clausi* and *A. tonsa* (Copepoda). *Limnol. Oceanogr. ***42**, 164–170. 10.4319/lo.1997.42.1.0164 (1997).

[19] Fields, D. & Yen, J. Fluid mechanosensory stimulation of behaviour from a planktonic marine copepod, *Euchaeta rimana* Bradford. *J. Plankton Res. ***24**, 747–755. 10.1093/plankt/24.8.747 (2002).

[20] Kiørboe, T. & Visser, A. Predator and prey perception in copepods due to hydromechanical signals. *Mar. Ecol. Prog. Ser. ***179**, 81–95. 10.3354/meps179081 (1999).

[21] Woodson, C., Webster, D. & True, A. Copepod behavior: Oceanographic cues, distributions and trophic interactions. In *Copepods: Diversity, Habitat, and Behavior, 215–253* (ed. Seuront, L.) (Nova Publishers, 2014).

[22] Yen, J. Directionality and swimming speeds in predator-prey and male-female interactions of *Euchaeta rimana*, a subtropical marine copepod. *Bull. Mar. Sci. ***43**, 395–403 (1988).

[23] Båmstedt, U. & Skjoldal, H. Studies on the deep-water pelagic community of Korsfjorden, Western Norway: Adenosine phosphates and nucleic acids in* Euchaeta norvegica* (Copepoda) in relation to its life cycle. *Sarsia. ***60**, 63–80. 10.1080/00364827.1976.10411296 (1976).

[24] Yen, J. Effects of prey concentration, prey size, predator life stage, predator starvation, and season on predation rates of the carnivorous copepod* Euchaeta elongata*. *Mar. Biol. ***75**, 69–77. 10.1007/BF00392632 (1983).

[25] Yen, J. Selective predation by the carnivorous marine copepod *Euchaeta elongata*: Laboratory measurements of predation rates verified by field observations of temporal and spatial feeding patterns. *Limnol. Oceanogr. ***30**, 577–597. 10.4319/lo.1985.30.3.0577 (1985).

[26] Catton, K., Webster, D. & Yen, J. The effect of fluid viscosity, habitat temperature, and body size on the flow disturbance of Euchaeta. *Limnol. Oceanogr. Fluids Environ. ***2**, 80–92. 10.1215/21573689-1894514 (2012).

[27] Catton, K., Webster, D., Brown, J. & Yen, J. Quantitative analysis of tethered and free-swimming copepodid flow fields. *J. Exp. Biol. ***210**, 299–310. 10.1242/jeb.02633 (2007). 17210966 10.1242/jeb.02633

[28] Adhikari, D., Webster, D. & Yen, J. Portable tomographic PIV measurements of swimming shelled Antarctic pteropods. *Exp. Fluids. ***57**, 180. 10.1007/s00348-016-2269-7 (2016).

[29] Wieneke, B. Volume self-calibration for 3D particle image velocimetry. *Exp. Fluids. ***45**, 549–556. 10.1007/s00348-008-0521-5 (2008).

[30] Adhikari, D. & Longmire, E. Visual hull method for tomographic PIV measurement of flow around moving objects. *Exp. Fluids. ***53**, 943–964. 10.1007/s00348-012-1338-9 (2012).

[31] Kiørboe, T., Saiz, E. & Visser, A. Hydrodynamic signal perception in the copepod *Acartia tonsa*. *Mar. Ecol. Prog. Ser. ***179**, 97–111. 10.3354/meps179097 (1999).

[32] Bundy, M. & Paffenhöfer, G. Analysis of flow fields associated with freely swimming calanoid copepods. *Mar. Ecol. Prog. Ser. ***133**, 99–113. 10.3354/meps133099 (1996).

[33] Tiselius, P. & Jonsson, P. Foraging behaviour of six calanoid copepods: Observations and hydrodynamic analysis. *Mar. Ecol. Prog. Ser. ***66**, 23–33. 10.3354/meps066023 (1990).

[34] Wolfgang, M., Anderson, J., Grosenbaugh, M., Yue, D. & Triantafyllou, M. Near-body flow dynamics in swimming fish. *J. Exp. Biol. ***202**, 2303–2327. 10.1242/jeb.202.17.2303 (1999). 10441083 10.1242/jeb.202.17.2303

[35] Drucker, E. & Lauder, G. Wake dynamics and fluid forces of turning maneuvers in sunfish. *J. Exp. Biol. ***204**, 431–442. 10.1242/jeb.204.3.431 (2001). 11171296 10.1242/jeb.204.3.431

[36] Mendelson, L. & Techet, A. Quantitative wake analysis of a freely swimming fish using 3D synthetic aperture PIV. *Exp. Fluids. ***56**, 135. 10.1007/s00348-015-2003-x (2015).

[37] Dabiri, J. et al. Jellyfish and fish solve the challenges of turning dynamics similarly to achieve high maneuverability. *Fluids. ***5**, 106. 10.3390/fluids5030106 (2020).

